# pK_a_ determination of oxysophocarpine by reversed - phase high performance liquid chromatography

**DOI:** 10.1186/2193-1801-2-270

**Published:** 2013-06-20

**Authors:** Huiling Huo, Tingting Li, Lei Zhang

**Affiliations:** College of Chinese Traditional Medicine, Guangzhou University of Traditional Chinese Medicine, 232 East of Waihuan Road, University Town, Guangzhou, Guangdong 510006 R.P. China; College of Chinese Traditional Medicine, Southern Medical University, Guangzhou, Guangdong 510515 R.P. China

**Keywords:** Oxysophocarpine, pK_a_ value, HPLC

## Abstract

**Background:**

In this study, a RP - HPLC method was applied for determination of pK_a_ value by using the dependence of the capacity factor (*k*) on the pH of the mobile phase for oxysophocarpine (OSP).

**Findings:**

The effect of the mobile phase composition on the ionization constant was studied by measuring the pK_a_ value at different MeOH concentrations, ranging from 10 to 20% (*v/v*). Based on all pH - *k* curves plotted and pH values at inflection point calculated, experimental pK_a_ value obtained for oxysophocarpine was 6.5.

**Conclusion:**

This method was successfully applied to realize low sample consumption, rapid sample throughput, high sensitivity and precision.

## Introduction

Oxysophocarpine (OSP) is one of the active alkaloid ingredients in the traditional Chinese medical herb kuh-seng (*Sophora flavescens* Ait.). Kuh-seng has a long history of curing cancer, viral hepatitis, cardiac arrhythmia, skin diseases and so on, and the curative effects have been proved definitely clear. Alkaloids, such as matrine, oxymatrine, sophocarpine and oxysophocarpine (OSP) (Lin et al. 2010), are commonly known to present in kuh-seng as main active constituents. Previous study found OSP could effectively adjust the immunity and antiviral system, but the reports about its physicochemical property was just a little, especially the pK_a_ value of OSP had not been determined.

To determine an ionization constant (pK_a_), is of great importance in many scientific areas, particularly in pharmacy. For instance, the lipophilicity, solubility and permeability of drug compounds are pK_a_ dependent, so it is important to obtain the reliable information about pK_a_ as early as possible during the drug development process (Wiczling et al. 2004). Factors talked above can define the fate of a drug in vivo, and the early-obtained information about drug absorption, distribution and transport processes may decrease costly the development process of a compound with poor pharmaceutical properties.

The commonly used techniques for pK_a_ determination are potentiometry, conductometry and spectrometry (Narasimham & Barhate 2011; Foulona et al. 2007; Gonen & Rytwo 2009). The first two methods are accurate, but both exist high sample consumption problem. Comparatively, UV spectrometry is being of relatively higher sensitivity which requires a big difference of UV spectrograms between a compound’s acidic (*HA*) and its basic (*A*) forms being observed. In our pre-experiment, spectrograms of OSP at different pH buffer solution were obtained, but the wavelength shifts as a function of pH was not obvious. As a result, the suitable detection wavelength could not be chosen.

Recently, some scholars successfully applied HPLC method for determination of the pK_a_ value for a compound (Padró et al. 2012; Demiralay et al. 2009; Wiczling et al. 2008; Wiczling et al. 2006; Pehourcq et al. 2006; Subirats et al. 2007; Miklautz et al. 2006). With analysis of the change in retention time (*t*_*R*_) of analyte versus the pH environment change of the mobile phase, Horvάth et al. (1997) proposed the theoretical basis for studying the pH dependence of chromatographic retention for ionizable solute in liquid chromatography. According to this theory, Angelov T et al. estimated the pK_a_ value of parabens by HPLC with a formula (1)  and its conversion formula (2)  (where *k* is the retention factor at a given pH of the compound investigated. *k*_*HA*_ and *k*_*A*_ are the retention factor of unionized and fully ionized forms, respectively), and the results showed the determined pK_a_ value was close to the literature values (Angelov et al. 2008). Wei Zhang et al. applied above method to determine the pK_a_ value of scutellarin. According to the retention factor of detective compound and corresponding pH values of mobile phase, they derived a curve equation of pH - *k* (Wei et al. 2010). Thanks to the curve fitting software, the pK_a_ value was determined as the inflection point in the curve.

Consequently, in the light of therapeutical value of OSP and the importance of the pK_a_ value for a compound, this paper firstly developed the HPLC method to obtain the pK_a_ value of OSP, and further investigated the effect of different percentage of organic phase in MeOH - phosphate buffer mixtures, 10, 15, 20% (*v/v*), on the pK_a_ value.

## Experimental

### Chemicals and reagents

The standard substance of oxysophocarpine (purity about 98%) was purchased from National Institute for the Control of Pharmaceutical and Biological Products (Beijing, China). HPLC grade methanol (Caledon Lab Ltd., Georgetown, Germany) was used for chromatographic separation. Phosphoric acid, sodium dihydrogen phosphate and disodium hydrogen phosphate were of analytical grade from Guangzhou Chemicals Co. Ltd. Deionized water used throughout the experiments was purified by Milli-Q system (Millilpore, Bedford, MA, USA).

### Apparatus

The HPLC analysis was carried out on a Shimadzu LC-20A System with N2010 chromatographic software, LC-20AT pump, SPO-M20A diode array detector, SIL-20A Auto sampler and CT0-10AS column oven. An Ultimate®XB-C_18_ column (4.6 × 250 mm ID, 5 μm ) was used for the determination.

Measurement of the pH value of the mobile phase was done with a PHB-3 pH electrode.

### Preparation of the phosphate buffers at different pH values

The different pH ranges of the phosphate buffers, between 2.0 to 9.5, were obtained by adjustment with sodium dihydrogen phosphate (0.01 mol/L), disodium hydrogen phosphate (0.01 mol/L) and phosphoric acid (0.01 mol/L).

### Chromatographic procedure

In this experiment, the mobile phases assayed were MeOH - phosphate buffer at 20:80 (*v/v*), 15:85 (*v/v*) and 10:90 (*v/v*), respectively, and pH values of each mobile phase were tested and summarized in Tables 1 and 2. The flow rate was maintained at 1.0 mL · min^-1^. For each OSP (50 μg · mL^-1^ in water), the retention time values (*t*_*R*_) were determined for every mobile phase composition and pH considered. Capacity factors were calculated as *k* = (*t*_*R*_ - *t*_*0*_) / *t*_*0*_. The dead time value (*t*_*0*_) was measured by injecting uracil (0.1%, in water). The chromatographic conditions were as follows: volume injected: 10 μL; column temperature: 25°C; UV detection wavelength: 210 nm.

**Table 1:** **The data of 20% (*v/v*) MeOH with calculation method**

No.	***pH*** of mobile phase	***t***_***R***_	Tailing factor of peak	***k***	***pH***_( ***i+*** 1)_–***pH***_***i***_	***k***_( ***i+*** 1)_– ***k***_***i***_	(***k***_( ***i+*** 1)_– ***k***_***i***_) / (***pH***_( ***i+*** 1)_–***pH***_***i***_)
1	2.3	5.322	1.288	0.844	-	-	-
2	2.6	5.328	1.279	0.846	0.3	0.002	0.007
3	3.9	5.594	1.308	0.938	1.3	0.092	0.071
4	5.1	6.236	1.493	1.161	1.2	0.223	0.186
5	6.2	8.748	2.802	2.031	1.1	1.870	0.791
6	7.1	15.978	4.367	4.536	0.9	2.505	2.784
7	**7.6**	**22.01**	**4.370**	**6.626**	**0.5**	**2.090**	**4.180**
8	8.0	22.755	4.408	6.885	0.4	0.258	0.645
9	9.1	23.219	3.945	7.045	1.1	0.161	0.145

**Table 2:** **The data of 15% (*v/v*) MeOH with calculation method**

No.	***pH*** of mobile phase	***t***_***R***_	Tailing factor of peak	***k***	***pH***_( ***i+*** 1)_–***pH***_***i***_	***k***_( ***i+*** 1)_– ***k***_***i***_	(***k***_( ***i+*** 1)_– ***k***_***i***_) / (***pH***_( ***i+*** 1)_–***pH***_***i***_)
1	2.2	8.091	1.305	1.804	-	-	-
2	2.5	8.320	1.294	1.883	0.3	0.079	0.264
3	3.9	8.733	1.338	2.026	1.4	0.143	0.102
4	5.0	9.881	1.665	2.424	1.1	0.398	0.362
5	6.0	15.076	3.319	4.224	1.0	1.800	1.800
6	**7.0**	**25.871**	**5.777**	**7.964**	**1.0**	**3.740**	**3.740**
7	7.6	29.759	5.792	9.312	0.6	1.348	2.247
8	7.9	30.805	5.834	9.674	0.3	0.362	1.207
9	8.9	31.549	5.826	9.932	1.0	0.258	0.258

### Precision evaluation of chromatographic system

Five successive sample injections to LC column eluted by different mobile phase, such as MeOH - phosphate buffer (pH6.0, which was close to the inflection point in pH - *k* plot) mixture at 20:80 (*v/v*), 15:85 (*v/v*) and 10:90 (*v/v*), respectively, were operated to verify the precision of the chromatographic system. Then the relative standard deviation (RSD) of retention times (*t*_*R*_) and peak areas (*A*) for OSP under different mobile phase conditions were calculated.

## Results and discussion

### Chromatographic condition and chromatogram behavior

The precision of chromatographic system was described in Table 3 that showed the RSD (%) values of retention times (*t*_*R*_) and peak areas (*A*) of OSP at each mobile phase conditions were all in normal range (<2%), indicating the chromatographic system was acceptable for determination of OSP.

**Table 3:** **The data of precision evaluation of chromatographic system (n=5, mean)**

No.	MeOH-phosphate	***pH***	***t***_***R***_	RSD (%)	***Area***	RSD (%)
1	20:80 (*v/v*)	6.2	8.750	0.08	1012394	1.08
2	15:85 (*v/v*)	6.0	15.034	0.20	969174	0.42
3	10:90 (*v/v*)	6.0	30.60	0.42	867097	1.39

**Figure 1:**
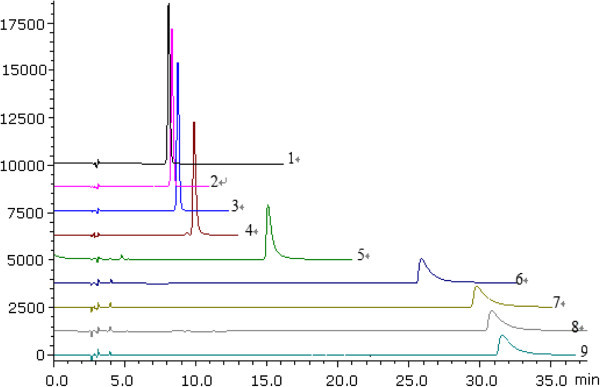
**The typical HPLC chromatograms of oxysophocarpine eluted by the mobile phase of 15% (*v/v*) MeOH.** (**1** = pH2.2, **2** = pH2.5, **3** = pH 3.9, **4** = pH 5.0, **5** = pH 6.0, **6** = pH7.0, **7** = pH7.6.7,**8** = pH7.9, **9** = pH8.9).

**Figure 2:**
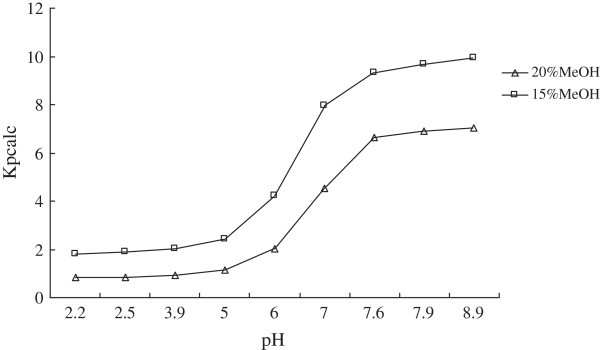
**The plot of capacity factors of OSP versus the pH of mobile phase for 20% (v/v) and 15% (v/v) MeOH.**

The effect of different mobile phase composition on the ionization constant was studied by measuring the pK_a_ values of OSP at different MeOH concentrations, ranging from 10% to 20% (*v/v*). The results showed that the mobile phase at 10% (*v/v*) MeOH was too weak to elute the compound when the pH >7.5. On the other hand, when the MeOH concentration more than 20% (*v/v*), the retention time (*t*_*R*_) of OSP at lower pH condition was close to the dead time (*t*_*0*_) value, which would effect the accuracy of the experimental data. Therefore, MeOH - phosphate buffer at 20:80 (*v/v*), 15:85 (*v/v*) were chosen to estimate the pK_a_ values of OSP in this study. Figure 1 showed the typical chromatograms of OSP eluted by MeOH - phosphate buffer (15:85) (*v/v*) at different pH environment. It provided the retention time (*t*_*R*_) of OSP would be increased as the pH value increased. The retention behavior of OSP under two different mobile phase conditions was summarized in Tables 1 and 2, and the plots of capacity factors (*k*) of OSP versus pH of the mobile phase were given in Figure 2.

### Data processing

In the pH - *k* plot of a compound investigated, the pH value at the inflection point in the curve, which is the equilibrium point for acidic (*HA*) and basic (*A*) forms of the compound, corresponds to the pK_a_ value of analyte (Angelov et al. 2008). Based on the formula (2) (Angelov et al. 2008) mentioned in above ‘Introduction’, this study ascertained the values (*k*, *pH*, *k*_*HA*_, *k*_*A*_) by finding the point with maximal slope in the sigmoidal curve.

The pH - *k* plot of OSP was a sigmoid curve, as the pH value increased, the change trend of capacity factor (*k*) was steady - sharp rise - steady, as shown in Figure 2. Through calculating the value of (*k*_*i+1*_– *k*_*i*_) / (*pH*_*i+1*_– *pH*_*i*_), the slope of specific detected points in the curve had been calculated, as shown in Tables 1 and 2. In the formula (2), the *pH* and *k* value were defined as the corresponding point with maximum-slope. The *k*_*HA*_ was the minimum value of the retention factor (*k*) , whose slope of corresponding point approximated zero, and similarly *k*_*A*_ was the maximum one.

In the Table 1, when using MeOH - phosphate buffer at 20:80 (*v/v*) as mobile phase, the *k*_*HA*_, *k*_*A*_, *pH* and *k* required by the formula were determined as 0.844, 7.045, 7.6 and 6.626 respectively, so the pK_a_ value of OSP was calculated as 6.46. In the Table 2, when using MeOH - phosphate buffer at 15:85 (*v/v*) as mobile phase, the *k*_*HA*_, *k*_*A*_, *pH* and *k* required by the formula were determined as 1.804, 9.932, 7.0 and 7.964 respectively, and the pK_a_ value of OSP was calculated as 6.50. From these result, the organic modifier percentage in the mobile phase had no influence on the pK_a_ detection. However, when the percentage of MeOH was lower than 10% (*v/v*), and the pH of mobile phase was above 7.5, OSP could not be eluted so as to hard to obtain a complete pH - *k* curve.

## Conclusion

In this study, the RP - HPLC method was successfully applied to determine the pK_a_ value of a compound investigated with low sample consumption, rapid sample analysis, high sensitivity and precision. Simultaneously, the ionization constant of OSP obtained from this test will play an important role in the field of pharmacokinetics and clinical treatment.
